# Acute Calcific Tendinitis of the Flexor Hallucis Brevis Associated With Muay Thai

**DOI:** 10.7759/cureus.68409

**Published:** 2024-09-01

**Authors:** Takeomi Nakamura, Yukinori Hara, Kazumasa Nishikata, Makoto Hodohara, Kenichi Kawano

**Affiliations:** 1 Orthopaedics, Tokyo Metropolitan Hiroo Hospital, Tokyo, JPN

**Keywords:** great toe pain, sesamoid bones, muay thai, athlete, inflammatory arthritis, acute calcific tendinitis, ultrasound, flexor hallucis brevis, plantar pain

## Abstract

Calcific tendinitis manifesting in the foot and toes is a rare condition that often goes unnoticed, even by podiatric specialists and healthcare practitioners. Characterized by an acute onset, this condition presents with pronounced local inflammatory indicators accompanied by pain, often complicating its differentiation from other conditions. We document our experience with a 27-year-old female patient presenting with calcific tendinitis in the flexor hallucis brevis (FHB), along with a review of the relevant literature.

## Introduction

Calcific tendinitis predominantly affects the tendons around the shoulder joint, with about 69-79% of cases occurring in this area [1]. Although this pathological manifestation can potentially arise in the periphery of any joint throughout the body, its occurrence within the foot and toes is exceptionally rare [2], accounting for roughly 1% of cases [3]. Previous literature on calcific tendinitis affecting the flexor hallucis brevis (FHB) is notably scarce [4], making its diagnosis often challenging. In this report, we detail our experience with a 27-year-old female patient exhibiting calcific inflammation of the FHB, necessitating careful differentiation from purulent arthritis of the hallux and sesamoid bone complications, all while drawing comparisons with previous literature findings focusing on pathophysiology.

## Case presentation

A 27-year-old female presented to our hospital complaining of plantar pain in her left great toe, which she had been experiencing since awakening three days prior. The patient's medical history indicated pyelonephritis four weeks prior, but she was otherwise healthy. She had begun attending Muay Thai classes three weeks earlier.

Her body mass index was 18.2, and vital signs revealed a temperature of 37°C with all other parameters within the normal range. The hallux was notably erythematous and edematous and exhibited severe pain. Redness was observed surrounding the metatarsophalangeal (MTP) joint of the hallux, which exhibited distinct characteristics upon passive dorsiflexion. Pain persisted even at rest and, at its peak, was rated at nine out of 10 on the numeric rating scale (NRS) [5]. A localized trigger point was identified medially on the toe, and even a slight extension of 3-5° provoked intense pain. Initial laboratory tests during her first consultation in our department showed a white blood cell (WBC) count of 9,200 /μL and a C-reactive protein (CRP) level of 0.39 mg/dL (Table 1). It was initially suspected that the patient had developed gout or pseudogout, but the uric acid levels were within the normal range.

**Table 1:** Summary of laboratory data results The white blood cell and C-reactive protein values were slightly elevated.

Test	Value	Reference range
White blood cell	9,200	3,300-8,600 /μL
Uric acid	4.6	2.6-5.5 mg/dL
C-reactive protein	0.39	0.00-0.14 mg/dL

**Figure 1:**
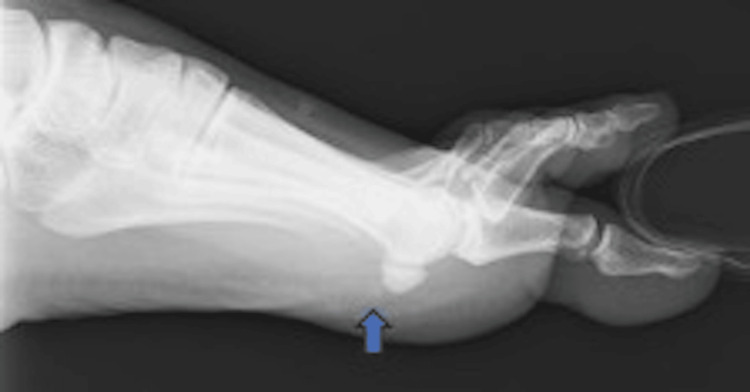
An initial X-ray The arrow shows a cloudy calcification adjacent to the medial sesamoid.

**Figure 2:**
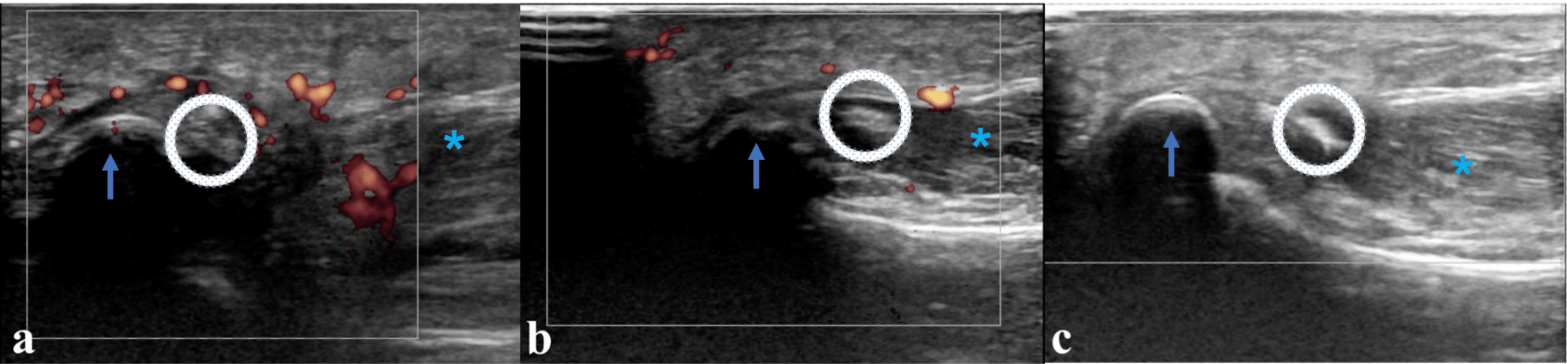
An ultrasound The arrows indicate the sesamoid bone. The asterisks indicate the flexor hallucis brevis. The blue circles indicate calcification. (a) Initial ultrasound: significant inflammation was visible around the calcification in the color Doppler imaging mode of the ultrasound. (b) Secondary follow-up. (c) Final follow-up: inflammatory changes were not observed during the final follow-up.

An X-ray of the toes was taken, which revealed a small fragment or calcification near the sesamoids (Figure 1). Ultrasonography detected calcific lesions, causing some acoustic shadowing, attached to the medial sesamoid bone of the FHB (Figure 2).

**Figure 3:**
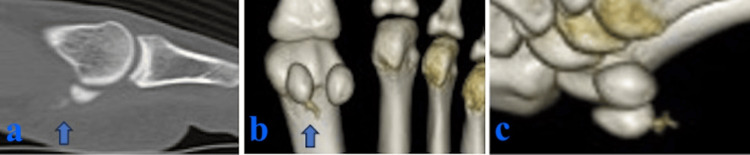
CT scans (a) Sagittal view. (b, c) 3D CT images. The arrows indicate calcification in the flexor hallucis brevis.

A pronounced Doppler effect was observed around the medial FHB. A computed tomography (CT) scan confirmed the continuity of the calcific lesion, measuring 5.2 × 3 mm, with the medial sesamoid bone (Figure 3). Treatment for calcific tendinitis consisted of oral nonsteroidal anti-inflammatory drugs (NSAIDs) (loxoprofen sodium hydrate 180 mg per day) and offloading with the aid of crutches.

Three days after the intervention, its pain had decreased, registering at three on the NRS, accompanied by a reduction in swelling.

**Figure 4:**
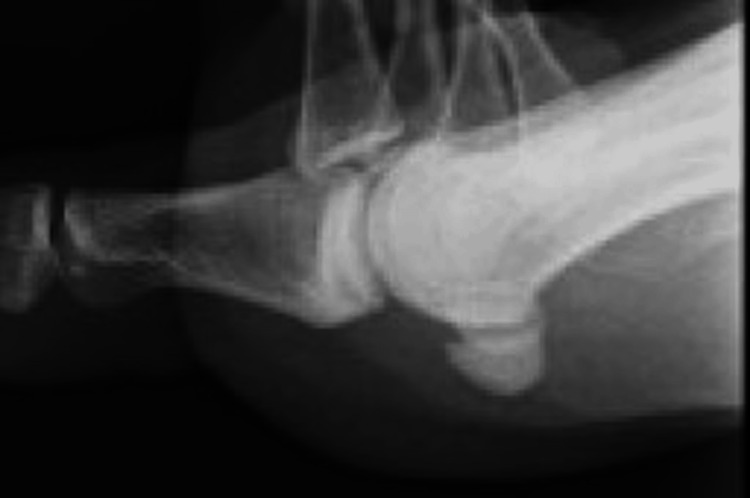
A final X-ray Calcification was no longer visible on the final X-ray.

Follow-up ultrasonography indicated a decrease in the inflammatory infiltration as observed in the Doppler. No purulent articular changes were detected on MRI. A week post-onset, the patient's resting pain had resolved, leaving only residual pain upon weight-bearing. By the second week, although some pain persisted during weight-bearing (NRS 1), swelling had entirely subsided, and blood tests showed no inflammatory signs. Repeated X-rays did not reveal any detectable calcific lesions (Figure 4), and only a faint trace was discernible upon ultrasonographic examination.

**Figure 5:**
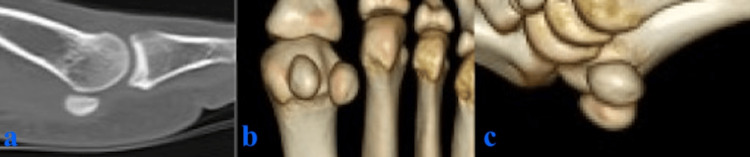
A final CT scan (a) Sagittal view. (b, c) 3D images. Calcification was completely resolved on the final follow-up.

The Doppler effect had completely vanished. Four weeks post-onset, the pain had entirely resolved, the range of motion of the hallux had completely returned to normal, and the calcification had completely resolved on CT scans (Figure 5). The patient's follow-up was concluded.

## Discussion

While calcific tendinitis typically affects the shoulder due to calcium hydroxyapatite build-up in a tendon, our case is less common. Calcific tendinitis of the foot is likely to manifest when it is subjected to repetitive external forces or excessive strain, as seen in sports.

The exact pathophysiology for developing this condition in the foot is still unclear. In this case, the onset can be attributed to exertion on the plantar aspect of the foot due to barefoot Muay Thai training. In a previously published report, Rowley investigated morphological differences between the flexor hallucis longus (FHL) and the FHB using intramuscular electromyography and reported that muscle activation during the toe-pushing task appeared to be more dependent on the FHB for most of the 20 subjects [6]. It is well known that Muay Thai involves multiple kicking techniques, which necessitates repeated toe-pushing movements.

Women are reportedly more susceptible to this condition than men [7]. Previously documented cases have been found in professions such as bus drivers, with the onset attributed to repetitive accelerator pedal operations [8]. Our patient was employed in automobile sales, a profession that entails significant driving duties. This mechanical stress resulting from the toe-pushing movement likely also contributed to the development of calcification.

In the literature, Hession reported a case of a 23-year-old female with calcific tendinitis of the FHB caused by practicing martial arts. The patient was treated successfully with an injection of steroid and Bupivacaine [9]. The most common treatment for this condition consists of rest, NSAIDs, and steroid injection [1,7]. Similar to calcific lesions of the shoulder, a suction method using a syringe also exists [7], but if not implemented promptly post-onset, there is a potential inability to aspirate the calcifications, as they are likely to become organized and stable. In this instance, improvement was observed with NSAIDs, negating the necessity for subsequent consultations. Literature suggests that calcifications tend to resolve within approximately two to three weeks. In our case, resolution was confirmed at four weeks post-onset.

Differentiation from purulent arthritis was challenging, but MRI examination and the course of progression ruled out this possibility. Septic arthritis of the MTP joint would be characterized by thick synovial enhancement, joint effusion, and varying degrees of cartilage destruction on MRI [7]. In instances of uncertain diagnosis, there is a risk of administering unnecessary antibiotics. In cases of sesamoid disorders, the Doppler effect is often absent. For follow-ups, ultrasound is highly useful due to its lower cost and lack of radiation exposure. In our case, the condition has improved, and the inflammatory infiltration has tended to disappear completely. While the prognosis is generally favorable, it is recommended that when examining post-exercise great toe pain in relatively young, middle-aged women, this condition should be considered and scrutinized in the differential diagnosis.

## Conclusions

This case highlights the importance of considering calcific tendinitis in the differential diagnosis of acute hallux pain. The 27-year-old female patient with calcific tendinitis in the FHB presented diagnostic challenges due to its similarities to other inflammatory conditions. Prompt diagnosis through careful evaluation and imaging studies such as ultrasound, CT scans, and MRI led to successful treatment with NSAIDs and conservative measures, resulting in a full recovery within four weeks.

This case underscores the potential link between repetitive physical activity that requires repeated toe-pushing movements, such as Muay Thai, and the development of calcific tendinitis in the foot. It also emphasizes the need to consider this condition in young, active women with unexplained foot pain, contributing valuable insights into its clinical presentation and management.
